# A Comparison of Measures of Endothelial Function in Patients with Peripheral Arterial Disease and Age and Gender Matched Controls

**DOI:** 10.1155/2016/2969740

**Published:** 2016-01-31

**Authors:** Richard B. Allan, Simon V. Vun, J. Ian Spark

**Affiliations:** ^1^Department of Vascular and Endovascular Surgery, Flinders University, Bedford Park, SA 5042, Australia; ^2^Department of Vascular and Endovascular Surgery, Flinders Medical Centre, Bedford Park, SA 5042, Australia

## Abstract

This study compared flow-mediated dilatation (FMD), peripheral artery tonometry (PAT), and serum nitric oxide (NO) measures of endothelial function in patients with peripheral artery disease (PAD) against age/gender matched controls. 25 patients (mean age: 72.4 years, M : F 18 : 7) with established PAD and an age/gender matched group of 25 healthy controls (mean age: 72.4 years, M : F 18 : 7) were studied. Endothelial function was measured using the % FMD, reactive hyperemia index (RHI) using PAT and serum NO (*μ*mol). Difference for each method between PAD and control patients and correlation between the methods were investigated. FMD and RHI were lower in patients with PAD (median FMD for PAD = 2.16% versus control = 3.77%, *p* = 0.034 and median RHI in PAD = 1.64 versus control = 1.92, *p* = 0.005). NO levels were not significantly different between the groups (PAD median = 7.70 *μ*mol, control median = 13.05 *μ*mol, *p* = 0.662). These results were obtained in elderly patients and cannot be extrapolated to younger individuals. FMD and PAT both demonstrated a lower hyperaemic response in patients with PAD; however, FMD results in PAD patients were unequivocally reduced whereas half the PAD patients had RHI values above the established threshold for endothelial dysfunction. This suggests that FMD is a more appropriate method for the measurement of NO-mediated endothelial function.

## 1. Introduction

The vascular endothelium, the functional lining of blood vessels, plays a critical role in vascular homeostasis. Through the local production of nitric oxide (NO) the normal endothelium has the capacity to regulate vascular tone, coagulation, inflammatory cell adhesion, and vascular smooth muscle cell proliferation [1, 2]. Whereas normal endothelial function is thought to be atheroprotective, endothelial dysfunction occurs early in the development of atherosclerotic lesions and is characterised by a prothrombotic phenotype and reduced bioavailability of nitric oxide [1, 3]. Indeed endothelial dysfunction is increased in atherosclerotic conditions such as coronary heart and peripheral artery disease (PAD) and is an independent predictor of future cardiovascular events [4, 5].

Given its central role in the pathogenesis of atherosclerosis, endothelial dysfunction is of great interest to investigators studying interventions that may improve endothelial function in the hope that improvements may modify disease progression and future cardiovascular risk. There is some evidence that endothelial dysfunction in patients with PAD [4, 6] can be improved; however, there are a number of different methods available to measure endothelial function and it is not clear which is the most appropriate for patients with PAD.

In a majority of clinical studies endothelial function is typically quantified by measuring flow-mediated dilatation (FMD), which is defined as vasodilation of an artery in response to an increase in luminal blood flow and thus laminar shear-stress [7]. This is routinely achieved by inducing hyperaemia following a brief ischaemic stimulus and vasodilatation occurs primarily through the effects of NO [8]. Measurement of FMD, however, is operator dependent and requires considerable skill and experience. Peripheral artery tonometry [9] (PAT) is a more recently developed alternative method of endothelial function measurement. PAT measures the ratio of baseline digital pulse wave amplitude at rest and after 5 minutes of brachial artery occlusion [10]. This method requires less training and experience and is largely automated making it an attractive alternative to FMD. While FMD and PAT both measure hyperaemic response, a surrogate for NO bioavailability, they are measured in different parts of the circulation (conduit and resistance vessels, resp.), with varying dependence on NO [11]. Furthermore, in our unit, we have observed a poor correlation between FMD and PAT [12].

Serum biomarkers have also been used to assess endothelial dysfunction; however, direct measurement of serum NO is problematic due to its highly reactive nature and short half-life. By using ELISA techniques to measure NO metabolites, nitrate, and nitrite, one can indirectly measure NO activity via serum or urine samples [13]; however, it remains unclear how this relates to other measures such as FMD and PAT.

While the above methods have previously been used to assess endothelial function in a number of different populations, a comparison of these three methods has not been performed in a group with PAD. The aim of this study was to investigate whether there was a difference between the PAD and control groups for each of the three measurement methods and whether there is a correlation between the methods.

## 2. Methods

### 2.1. Participant Selection

Data was collected from participants enrolled in two studies in which FMD, PAT, and venous blood collection were performed as part of the investigation of specific interventions.

The first study was investigating the effects on endothelial function of moderate dose fish oil on healthy participants with no evidence of PAD [14]. The second study was investigating the effects of two different exercise regimes on participants with intermittent claudication [15]. Baseline FMD and PAT tests and venous blood as part of these studies were used.

The sample populations consisted of 25 participants with symptomatic PAD (claudication, Rutherford classification 1–3) in the exercise intervention study and 25 controls, age and gender matched to the PAD group, from the fish oil intervention study. The age, gender, and BMI data for each group are presented in Table 1. The control group and PAD group were well matched with no significant difference for age, gender, or BMI between the groups. No participants were on nitrate-based medications. All patients with PAD were on statins (which were withheld for a 24-hour period prior to testing) while none were in the control group.

This analysis was limited to the baseline tests because of the nonindependent nature of follow-up measurements and the risk of confounding variables due to potential differing effects of the interventions on each test.

This study was approved by the Southern Adelaide Clinical Human Research Ethics Committee. All participants provided written informed consent for the measurement of FMD, PAT, and serum NO levels prior to commencement of data collection.

### 2.2. Peripheral Arterial Tonometry

Participants were instructed to fast for eight hours, refrain from caffeine, alcohol, and tobacco, and avoid exercise for eight hours prior to testing.

PAT tests were performed using an EndoPAT device (Itamar Medical Ltd., Caesarea, Israel), following the manufacturer's guidelines, in a quiet, dimmed, and temperature controlled room as previously described [12] with upper arm occlusion at 250 mmHg. The hyperaemic response was analysed using the device's proprietary software and the reactive hyperemia index (RHI) was calculated from pre- and post-occlusion measurements by the device software using the method previously described by McCrea et al. [10].

### 2.3. Brachial Artery Ultrasound Flow-Mediated Dilatation

Fifteen minutes after the completion of the PAT testing, FMD was performed by a single experienced operator with the participant supine and the right arm in a supportive cradle with the transducer held in a stereotactic stand, following accepted guidelines [16] using forearm occlusion as previously described [12]. Thirty seconds of baseline imaging and three minutes of post occlusion imaging were recorded to obtain baseline and the maximal hyperaemic response measurements. The brachial artery diameter was measured, during diastole, using automated edge detection software (Brachial Artery Analyser, MIA-LLC, Coralville, USA). The % FMD was calculated using the following equation:

(1) FMD%=maximum diameter −baseline diameterbaseline diameter×100.

### 2.4. Serum NO

Peripheral venous blood was drawn into 4 mL serum separator evacuated tubes, allowed to clot for 10 min, and then immediately centrifuged at 3,000 g for 7 minutes at 20°C. Serum was stored at −80 degrees Celsius and thawed for later total NO assay. Due to the instability and short half-life of NO, measurement was performed on the NO metabolites, nitrite (NO_2_^−^), and nitrate (NO_3_^−^). Nitrate was converted to nitrite using an enzyme nitrate reductase and the nitrite levels were then measured with a coloured azo dye product of the Griess reaction using visible light at 540 nm (Total Nitric Oxide Assay Kit; Pierce Biotechnology, Rockford, IL) [17]. The nitrite levels detected represented the total NO metabolites present in each sample and the kit is able to recover 98% of nitrate in serum. Results were expressed in *μ*mol with a detection limit of 0.35 *μ*mol and a CV of 5.3%.

### 2.5. Testing of Reproducibility

Test-retest reproducibility testing was performed for both FMD and PAT as described previously [12]. Brachial artery diameter intraclass coefficient (ICC) = 0.989 (*p* < 0.001) and coefficient of variation (CV) = 1.52%, FMD ICC = 0.884 (*p* = 0.002) and CV = 15.0%, and RHI ICC = 0.298 (*p* = 0.304) and CV = 19.3%. Intra-assay CV for serum total NO assays was 9.8%.

### 2.6. Statistical Analyses

Data were analysed using the SPSS for Windows statistical package version 20 (SPSS Inc., Chicago, IL, USA).

The mean, standard deviation (SD) and range were reported for age and body mass index (BMI) and Student's *t*-test was used to test for difference between controls and patients with PAD. Due to the nonparametric nature of the FMD, RHI, and serum NO results, data median and interquartile range (IQR) were reported and Mann-Whitney *U* tests were performed to assess for differences in results between the controls and those with PAD.

Correlation between FMD, RHI from PAT, and NO was assessed using Spearman correlation coefficient.

Test-retest reproducibility was assessed using the intraclass correlation coefficient (ICC) and coefficient of variation (CV) for the first and second measurements in the group of healthy volunteers.

All tests were two-tailed and the level of statistical significance was set at *p* < 0.05.

## 3. Results and Discussion

### 3.1. Results

**Figure 1:**
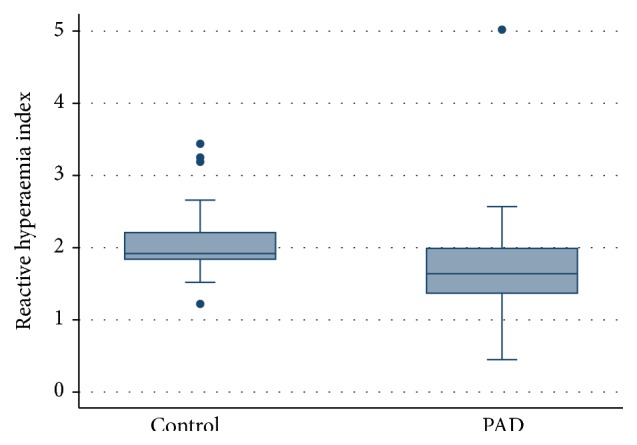
Plot of distribution of RHI values for age and gender matched controls and patients with PAD, *p* = 0.005.

**Figure 2:**
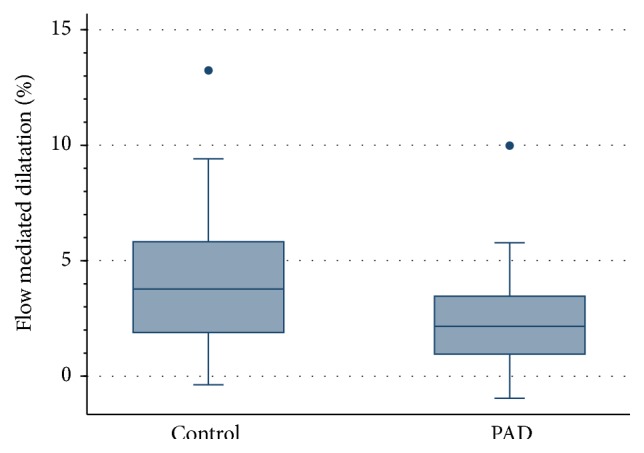
Plot of distribution of FMD % values for age and gender matched controls and patients with PAD, *p* = 0.034.

**Figure 3:**
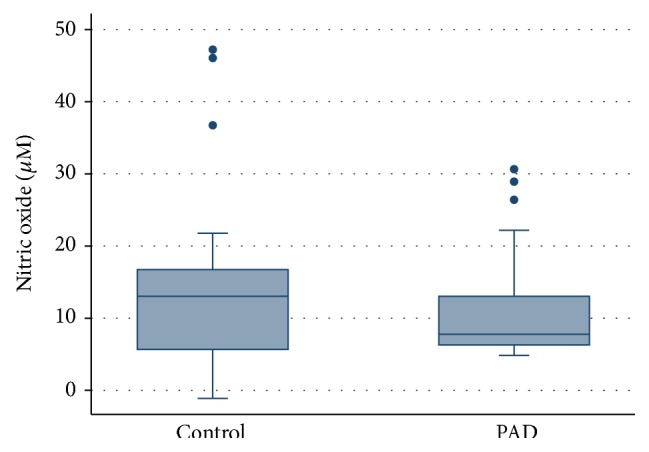
Plot of distribution of NO concentrations for age and gender matched controls and patients with PAD, *p* = 0.662.

The results for each test of endothelial function are presented in Table 2. The distribution of results for each method in controls and patients with PAD are displayed in Figures 1, 2, and 3.

FMD and RHI were significantly lower in PAD patients compared to controls (FMD *p* = 0.034; PAT *p* = 0.005). There was no significant difference in NO levels between the two groups (*p* = 0.662).

No correlation was seen between the three tests of endothelial function in the participants with PAD (RHI versus FMD: *r* = 0.182, *p* = 0.205, RHI versus NO: *r* = 0.034, *p* = 0.815, FMD versus NO: *r* = 0.07, *p* = 0.627).

### 3.2. Discussion

To the best of our knowledge this is the first study to investigate the relationship of FMD, PAT, and serum NO metabolites in a sample of patients with PAD and to compare these three measures of endothelial function between patients with PAD and a group controlled for age and gender. Both FMD and PAT measurements were significantly lower in this sample of PAD patients compared to age and gender matched controls; however, no significant difference was seen in results for serum NO metabolites.

The FMD results from the current study were markedly lower than that of the two previous studies of FMD in PAD patients and controls (5.40%–6.45% for PAD and 9.79%–12.80% for controls [3, 14] compared to 2.16% for PAD and 3.77% for controls in the current study). In part this may be due to differences in reporting of the data but it is also likely to be due to variations in technique as the two earlier studies used upper arm occlusion for FMD. This is known to be less dependent on NO-mediated dilatation and to produce a higher percentage dilatation [18] than the more widely accepted forearm method used in the current study. The current study is the first to demonstrate lower RHI in a sample of PAD patients compared to age and gender matched controls, indicating that the hyperaemic response is blunted in this patient group. However, the RHI measured in many of the PAD patients was high in relation to the previously reported threshold RHI value for endothelial dysfunction of 1.67 [9]. The median RHI of this group was 1.64 and based on the above threshold nearly half the PAD patients would be classified as having normal endothelial function. This is very surprising since this was a group with demonstrated symptomatic atherosclerotic disease and markedly lowered FMD values. It is known that NO-mediated vasodilatation is responsible for less than half the hyperemic response measured by PAT [19]. The relatively high RHIs measured by PAT in the PAD patients may relate to retention of the non-NO-mediated mechanisms that contribute to the hyperemic response as measured by PAT. This study highlights the need for studies to establish the underlying mechanisms of the hyperemic response in patients with PAD. These findings raise concerns that PAT may not be the most appropriate method for assessing NO-mediated endothelial function.

The lack of correlation between FMD and PAT in the current study is consistent with previous studies that use forearm occlusion for the FMD testing [12]. This is consistent with the evidence that these methods measure different aspects of the hyperemic response [18, 19].

The lower endothelial function found in PAD patients implies that there is the potential for improvement in endothelial function and this has been demonstrated in studies using FMD before and after surgical and endovascular interventions [20, 21]. The low FMD levels in the healthy controls (consistent with the known reduction in endothelial function with age [22]) suggest that the capacity for improvement in PAD patients may be limited and it is unclear if such mild improvements confer any long term health benefits related to reduced cardiovascular risk or delayed disease progression.

The lack of difference in serum measurement of NO metabolites may be due to the wide distribution in the NO results. This test is known to be highly sensitive to environmental factors [13]. Handling and analysis of samples were standardized in an effort to minimize these effects. In addition serum NO levels are affected by degradation associated with oxidative stress, which is expected to be higher in elderly patient with PAD. The high variability in results suggests that this may not be a robust test and may be of limited use in clinical studies.

A further limitation of this study is the variability of test results with all three methods. High variability of the NO results was found even with strict standardization of handling and analysis. FMD was found to have a better ICC and a CV than PAT. This is consistent with the only other report comparing reproducibility of the two methods [23]. It is well recognized that both of these methods have considerable test-retest variability. This appears to be intrinsic to the methods and relates to physiological variations rather than technical factors [24].

The use of vasoactive drugs is a potential source of variability; however, none of the participants were taking nitrate-based pharmacotherapy. All the PAD patients were on statins which are known to improve endothelial function [25]. As this study has demonstrated a reduction in endothelial function in these patients, the use of statins may, at worst, have decreased the observed reduction without affecting the overall conclusions.

A limitation of this study is that the endothelium-independent response of subjects was not assessed. This is feasible with FMD and allows assessment of both endothelial and nonendothelial mechanisms that may affect the hyperemic response. It is not feasible with PAT [26] and so comparison between these techniques could only be undertaken for endothelial-dependent vasodilatation.

Another potential limitation is that the participants were not recruited specifically for this study but were already enrolled in existing intervention studies. As data were restricted to preintervention baseline testing this should not have an influence on the reported results.

The small sample size of this study is also a limitation. However, even with this limitation there was a significant difference in FMD and RHI between PAD patients and controls.

## 4. Conclusion

This study has found that assessment of the hyperemic response by both FMD and PAT testing shows lower endothelial function in PAD patients when compared to age and gender matched controls. Serum NO results show a high degree of variation and did not demonstrate a significant difference between the groups. The difference in endothelial function between PAD patients and age-gender matched controls suggest an opportunity to improve endothelial function in PAD patients, though this may be limited by age dependent deterioration. Investigation is required into whether an improvement in endothelial function results in longer term health benefits in these patients.

An important limitation of these results that needs to be considered with this study is that the results were obtained in elderly patients with PAD and cannot be extrapolated to younger or less diseased individuals.

Both FMD and PAT are valid methods for assessing endothelial function in a research setting but the documented intertest variation makes them unsuitable for use in assessing individual patients in a clinical setting. If NO-mediated endothelial function is the phenomena of interest under investigation FMD would appear to be the more appropriate test as it is more purely NO-mediated in nature, demonstrates a more clear-cut reduction in patients with PAD, and has better reproducibility.

## Figures and Tables

**Table 1:** Characteristics of the sample groups.

	Controls (*n* = 25)	PAD (*n* = 25)	
Mean age (years) (SD), range	72.40 (7.21), 59–85	72.36 (8.91), 58–90	*p* = 0.986
Gender: male : female	7 : 18	7 : 18	
Mean BMI (SD), range	28.7 (5.19), 19.9–43.7	28.3 (5.18), 22.3–43.6	*p* = 0.803

PAD: peripheral arterial disease, SD: standard deviation, and BMI: body mass index.

**Table 2:** RHI, FMD, and NO results for age and gender matched controls and patients with PAD.

	Controls (*n* = 25)	PAD (*n* = 25)	
RHI median, IQR	1.92, 1.84–2.24	1.64, 1.34–2.01	*p* = 0.005
FMD% median, IQR	3.77, 1.87–6.15	2.16, 0.59–4.17	*p* = 0.034
NO (*μ*mol) median, IQR	13.05, 5.58–19.06	7.70, 6.17–15.07	*p* = 0.662

PAD: peripheral arterial disease, IQR: interquartile range, FMD: flow-mediated dilatation, RHI: reactive hyperaemia index, and NO: nitric oxide.
